# Aktuelles zu chirurgischen Therapieoptionen bei chronisch-entzündlichen Darmerkrankungen

**DOI:** 10.1007/s00108-024-01846-5

**Published:** 2025-01-30

**Authors:** Werner Kneist

**Affiliations:** Klinik für Allgemein‑, Viszeral- und Thoraxchirurgie, Klinikum Darmstadt GmbH, Grafenstraße 9, 64283 Darmstadt, Deutschland

**Keywords:** Robotisch assistierte chirurgische Verfahren, Restaurative Proktokolektomie, Morbus Crohn, Colitis ulcerosa, Chirurgische Anastomose, Robotic surgical procedures, Proctocolectomy, restorative, Crohn’s disease, Colitis, ulcerative, Anastomosis, surgical

## Abstract

Im Bereich der chirurgischen Behandlung chronisch-entzündlicher Darmerkrankungen (CED) gibt es national und international Leitlinien und Entwicklungen, die zu einer besseren Versorgung der Patienten beitragen. Die wichtigen Empfehlungen beziehen sich zunehmend auf individualisierte und minimal-invasive Ansätze mit Integration neuer Techniken. Die Indikation zur abdominellen Operation bleibt an eine Spezialisierung gebunden, nicht zuletzt um Sequenztherapie und Multimodalität in ihrer Bedeutung für die Risikominimierung und Ergebnisverbesserung auch weiterhin einschätzen zu können. In der vorliegenden Arbeit sollen zentrale chirurgische Aspekte der CED Therapie kurz vorgestellt und aktuell eingeordnet werden.

**Video online:**

Die Online-Version dieses Beitrags (10.1007/s00108-024-01846-5) enthält ergänzendes Videomaterial.

## Abstract (en)

There are national and international guidelines and developments for the surgery of chronic inflammatory bowel disease (IBD) that contribute to better patient care. Important recommendations include increasingly individualized and minimally invasive approaches with the integration of new technologies. The indication for abdominal surgery remains tied to specialization, not least in order to continue to be able to assess the importance of sequential treatment and multimodality in improving surgical results and minimizing risks. This paper aims to briefly present key surgical aspects and classify them according to the current state of knowledge.

Trotz einer zunehmenden Zahl medikamentöser und komplementärer Therapien bleibt die Operation eine tragende Säule in der Behandlung von Patienten mit chronisch-entzündlichen Darmerkrankungen (CED). Etwa bis zu einem Drittel der Patienten mit CED benötigen eine chirurgische Mitbehandlung. Die idealerweise einer Spezialisierung unterworfene operative Behandlung wurde in den letzten Jahren zunehmend minimal-invasiv durchgeführt.

Die Empfehlungen zur Diagnostik und Therapie und die sich daran anschließende Patientenaufklärung sind an die Diskussion in einem CED-Board gebunden und führen damit idealerweise zur partizipativen Entscheidungsfindung. Entwicklungen in der Chirurgie sollten dabei Beachtung finden. Sie werden ja immer auch in der Praxis bewertet. In der vorliegenden Arbeit werden aktuelle Aspekte aus viszeralchirurgischer Sicht kurz vorgestellt und eingeordnet.

## Geschlecht

Eine aktuelle Metaanalyse zeigt anhand von 14 gepoolten Studien, dass Männer mit CED signifikant häufiger operiert werden als Frauen [1]. Differenziert nach Erkrankung bleibt dieser Unterschied für Patienten mit Colitis ulcerosa (CU) signifikant (Odds Ratio [OR] 1,8; 95 %-Konfidenzintervall [KI] 1,2–2,7; *p* = 0,02) und bestätigt sich als Trend für Patienten mit Morbus Crohn (MC; OR 1,3; 95 %-KI 0,9–1,9; *p* = 0,10). Die durchaus geschlechtsabhängigen Ängste vor Infertilität, einem veränderten Körperbild und dem kolorektalen Karzinom dürfen bei einer chirurgischen Vorstellung nicht unterschätzt werden. Im Rahmen der partizipativen Entscheidungsfindung sind sie anzusprechen.

## Robotik

**Abb. 1:**
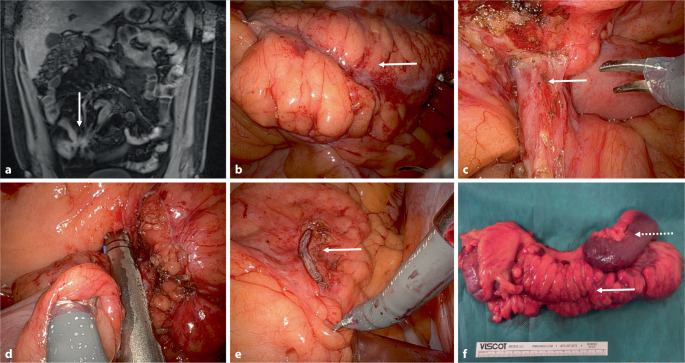
Robotisch assistierte Resektion bei 24-jährigem Patienten mit fistulierendem M. Crohn (Erstdiagnose 13 Jahre zuvor, medikamentöse Therapie zuletzt mit Infliximab, dann Vedolizumab). **a** Ileoileale Fistel im CT (*Pfeil*), **b** Ileitis terminalis, **c** auspräparierte ileosigmoidale Fistel (*Pfeil*), **d** Sigma-Wedge-Resektion der Fistel mit Klammernahtstapler, **e** Klammernaht am Sigma (*Pfeil*), **f** erweitertes Ileozökalresektat mit 30 cm Ileum incl. Loop der oralseitigen fisteltragenden Ileumschlinge (*gestrichelter Pfeil*) und sichtbarer Klammernaht nach Sigma-Wedge-Resektion (*Pfeil*)

Mit zunehmender Erfahrung und guten Ergebnissen wurde der minimal-invasive laparoskopische Zugang zur operativen Behandlung geeigneter Patienten mit CED akzeptiert; heute bildet er den Standard [2–4]. Spezialisierung führte bereits vor zehn Jahren zu einer Rate der minimal-invasiven Chirurgie (MIC) von 80 %, wobei ein Drittel dieser Fälle durchaus komplex war (Fisteln, Abszesse, Rezidive; [5]). In der letzten Dekade wurde das robotisch assistierte minimal-invasive Vorgehen zunehmend angewendet und wird zukünftig wohl für bestimmte viszeralchirurgische Operationsindikationen zur Methode der Wahl [6]. Die technischen Vorteile der Robotik gegenüber der Laparoskopie sind vor allem die verbesserten Bewegungsabläufe, ruhigere 3D-Sicht, Filterung von körperlichem Zittern und die im Vergleich mit der konventionellen Laparoskopie kürzere Lernkurve. Unabhängig von Kostenfragen und unbekannten Risiken ergeben sich durch moderne technische Schnittstellen somit auch für die CED Chirurgie immer auch neue Chancen. Erste systematische Aufarbeitungen der Literatur zu den Ergebnissen der robotischen CED-Chirurgie zeigten deren Machbarkeit und Sicherheit. Es ergaben sich Hinweise auf einen reduzierten Blutverlust, eine kürzere Krankenhausverweildauer sowie tendenziell niedrigere Komplikationsraten [7, 8], die durchaus nachvollziehbar sind (Abb. 1).

Gemäß US-amerikanischen Registerdaten erfolgen minimal-invasive Proktektomien zunehmend robotisch

**Abb. 2:**
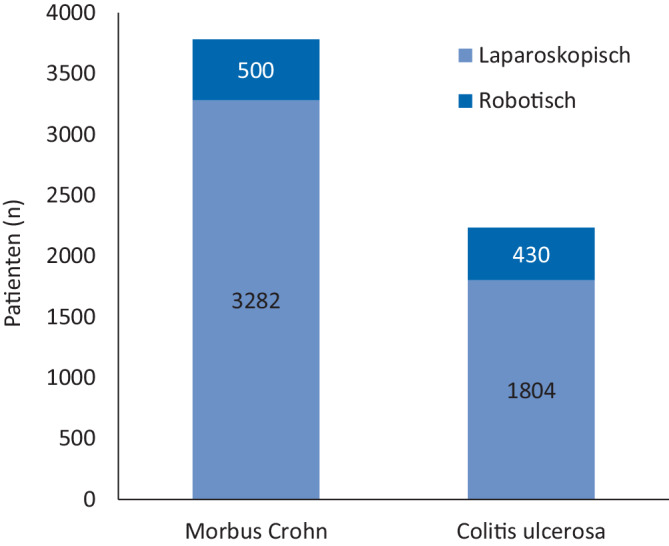
ACS-NSQIP-Datenbankauswertung zur minimal-invasiven Chirurgie (Kolektomie/Proktektomie) bei chronisch-entzündlicher Darmerkrankung 2019–2021 [9]. *ACS NSQIP* American College of Surgeons National Surgical Quality Improvement Program

Analysen US-amerikanischer Registerdaten ergaben für minimal-invasiv operierte Patienten einen signifikanten Anstieg der robotisch durchgeführten Proktektomien bei CU von 17,7 % im Jahr 2016 auf 41,0 % im Jahr 2021, bei MC von 14,3 % auf 35,8 % [9]. Für Kolonresektionen galt der gleiche Trend (von 3,2 % auf 12,4 % und von 4,0 % auf 12,2 %). Die multivariate Analyse von 6016 operierten Patienten (Abb. 2) ergab bezüglich der ungeplanten Konversion zum offenen Vorgehen und der Gesamtmorbidität weder bei CU noch bei MC eine Abhängigkeit vom MIC-Zugang. Bei Patienten mit MC wurden im Rahmen eines robotischen Verfahrens aber signifikant häufiger schwerere infektiöse Komplikationen (Clavien-Dindo-Grad III–IV) registriert (11,2 % vs. 7,3 %; OR 1,5; 95 %-KI 1,0–2,2; *p* = 0,03). Dies wurde zum Teil auf eine fehlende haptische Wahrnehmung des morphologisch veränderten Gewebes und auf verzögerte Operationen in der Coronavirus-disease-2019(COVID-19)-Pandemie zurückgeführt. Dennoch war die Robotik in dieser Gruppe mit signifikant weniger ungeplanten Konversionen (1,6 % vs. 3,8 %) und mit früheren Entlassungen (3. vs. 4. postoperativer Tag; *p* < 0,001) verbunden.

**Abb. 3:**
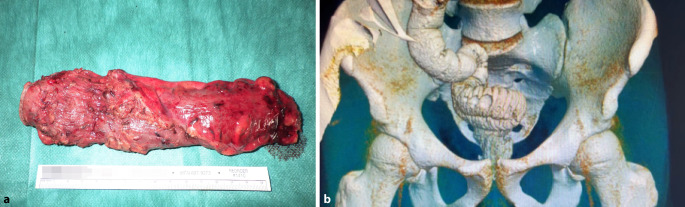
**a** Rektosigmoideales Präparat, **b** 3D-Rekonstruktion des CTs zur Pouchdarstellung und Dichtigkeitsprüfung vor geplanter Ileostomarückverlagerung (dreizeitige, robotisch assistierte Proktokolektomie mit intraoperativem, pelvinem Neuromonitoring (pIONM) bei 18-jährigem Patienten mit Colitis ulcerosa (siehe auch 3D-Darstellung des ileoanalen J-Pouches im Kurzvideo im elektronischen Zusatzmaterial online)

In einer aktuellen Metaanalyse wurden die Ergebnisse von 11 nichtrandomisierten Vergleichsstudien zusammengefasst (2 zur subtotalen Kolektomie; 3 zur Ileozökalresektion und 6 zur restaurativen Proktokolektomie; [10]). Von 5566 Patienten mit CED wurden 365 robotisch (6,6 %) und 5201 konventionell laparoskopisch operiert. Die robotisch assistierten Operationen waren insgesamt mit signifikant weniger Gesamtkomplikationen verbunden (OR 0,48; 95 %-KI 0,24–0,93; *p* = 0,03), dauerten im Mittel 41 min länger (*p* = 0,00001) und führten tendenziell zu einer kürzeren Krankenhausverweildauer (für subtotale Kolonresektion auch signifikant mit *p* = 0,03; Tab. 1).

**Tab. 1:** Ergebnisse einer Metaanalyse zur minimal-invasiven Chirurgie bei chronisch-entzündlicher Darmerkrankung [10]

	Studien (*n*)	Robotik	Laparoskopie
*Morbidität (%)*	10	30,3*	43,1*
Proktokolektomie (Pouch)	–	31,9	53,2
Ileozökalresektion	–	9,3	14,4
Subtotale Kolonresektion	–	42,1	44,0
*Ungeplante Konversion (%)*	8	4,0	11,2
*Anastomoseninsuffizienz (%)*	6	2,3	2,9
*Paralyse (%)*	7	22,9	22,9
*Wundinfekt (%)*	7	0,8	0,9
*Revisionsoperation (%)*	9	5,1	3,9
*Wiederaufnahme (%)*	4	12,9	20,7

*p < 0,05

## Ileozökalresektion

Die Ileozökalresektion ist die häufigste intestinale Operation bei MC-Patienten. Über die Wahl der Therapie (Operation vs. medikamentöse Therapie) gibt es jedoch weiterhin Debatten bei einer insgesamt sehr heterogenen klinischen Praxis. Der Expertenkonsens wurde aktuell (2023) überprüft und stellt weiterhin fest, dass zur Behandlung von MC-Patienten mit isoliertem Befall der Ileozökalregion, kurzer Krankengeschichte und fehlendem Ansprechen auf Steroide eine Ileozökalresektion der medikamentösen Therapie mit Infliximab gleichwertig gegenübersteht [4].

Mit einem Langzeit-Follow-up von 63,5 Monaten im Median (Interquartilsabstand 39,0–94,5 Monate) zeigte die LIR!C-Studie zum Vergleich von Anti-Tumornekrosefaktor(TNF)-Therapie und Ileozökalresektion, dass die Hälfte der mit Infliximab behandelten Patienten doch eine Operation benötigte und die restlichen Patienten weiterhin eine biologische Therapie. Bei den Patienten, die einer Ileozökalresektion unterzogen wurden, erfolgte keine weitere Operation und fast die Hälfte benötigte innerhalb von 5 Jahren auch keine andere Behandlung [11]. In Dänemark wurden landesweite Registerdaten von Patienten ausgewertet, die sich zwischen 2003 und 2018 einer Ileozökalresektion (581 Patienten; 24,8 % laparoskopisch) bzw. einer Anti-TNF-Therapie (698 Patienten) als Primärbehandlung unterzogen. Das Langzeit-Follow-up ergab, dass das kombinierte Risiko (kombinierter Endpunkt) einschließlich Krankenhausaufenthalt, wiederholter MC-bedingter Operation, systemischer Kortikosteroidexposition und perianaler MC-Erkrankung bei primär operierten Patienten um 33 % niedriger war als bei initialer Anti-TNF-Therapie. Etwa die Hälfte der Patienten mit Resektion (47 %) war nach 5 Jahren Nachbeobachtung ohne spezifische Therapie [12]. Mit dem Endpunkt „Vermeidung der Chirurgie“ zeigte eine aktuelle Metaanalyse für Patienten mit MC, dass eine frühere Therapie mit Biologika (innerhalb 3 Jahren nach Erstdiagnose oder Top-down-Behandlung) im Vergleich zu einer späteren Behandlung (> 3 Jahre nach Erstdiagnose oder Step-up-Behandlung) erfolgreicher ist (OR 0,63; 95 %-KI 0,48–0,84; *p* = 0,001). Die mittleren Nachbeobachtungszeiten der eingeschlossenen Studien lagen zwischen 12 und 103 Monaten [13].

In der Praxis sollte sich am bestmöglichen funktionellen Langzeitergebnis für die Patienten orientiert werden (und nicht auf die Vermeidung bestimmter Therapieformen). Empfehlungen sollten gemeinsam nach Fallbesprechung in einem interdiszplinären CED-Board dokumentiert werden. Danach kann auf die „kluge“ Empfehlung des CED Boards verwiesen und die Patientenpräferenz zur partizipativen Entscheidungsfindung berücksichtigt werden.

## Erweiterte mesenteriale Resektion

Bei Morbus Crohn ist die Schleimhaut oft transmural befallen, mit Beteiligung des Mesenteriums, intraoperativ sichtbar als dem Dünndarm angelagertes „creeping fat“. Um Rezidive des M. Crohn zu vermindern, wurden daher erweiterte mesenteriale Resektionstechniken für Dünndarmoperationen entwickelt. Retrospektive Daten deuteten darauf hin, dass die mesenterial erweiterte (darmferne) ileokolische Resektion bei M. Crohn mit einer Reduktion reoperationspflichtiger Rezidive einhergeht [14]. Die Befürchtung, dass durch eine zentralere (darmwandnahe) mesenteriale Dissektion mehr Komplikationen entstehen, scheint sich nicht zu bestätigen. Auf Basis der Datenbank des American College of Surgeons National Surgical Quality Improvement Program (ACS-NSQIP) wurde der frühe postoperative Verlauf von 3709 Patienten mit MC untersucht. Im Zeitraum von 2014 bis 2019 erhielten 622 (16,8 %) dieser Patienten eine erweiterte mesenteriale Resektion unter Einschluss von > 12 Lymphknoten (Surrogat). Weder für einzelne chirurgische Komplikationen (Blutung, Anastomoseninsuffizienz, Paralyse, Sepsis, Reoperation) noch für einen Composite-Score oder die Gesamtmorbidität ergab sich ein höheres Risiko gegenüber den weniger ausgedehnten Resektionen [15].

Jetzt zeigte die aktuell publizierte randomisierte, multizentrische SPICY-Studie, dass 6 Monate nach Ileozökalresektion kein Unterschied zwischen den Gruppen hinsichtlich der endoskopischen Rezidivrate besteht: 28/66 Patienten mit erweiterter Mesenterialresektion vs. 28/65 Patienten mit darmnaher Resektion (OR 0,99; 95 %-KI 0,66–1,46; *p* = 1,0). Die Rate postoperativer Komplikationen (Clavien-Dindo-Grad ≥ III) lag nach erweiterter Resektion bei 11 %, nach mesenteriumsparender Dissektion bei 8 % [16]. Weitere Studien widmen sich der Fragestellung [17]. Zunächst kann der darmwandnahen Resektion auch weiterhin der Vorzug gegeben werden.

## Kono-S-Anastomose

Nach der Ileozökalresektion bei MC gehören End-zu-End‑, End-zu-Seit‑, Seit-zu-Seit- und Kono-S-Anastomosen zu den geläufigen Techniken. Es kann maschinell geklammert und genäht werden. Für den methodischen Vergleich wird oft die endoskopische Rezidivhäufigkeit nach ≥ 6 Monaten herangezogen. In der aktuellen Metaanalyse deuten die gepoolten Raten auf signifikante Vorteile der Kono-S-Anastomose (*n* = 369) gegenüber den anderen Techniken (*n* = 2087) hin: 24,7 % (95 %-KI 6,8–49,4 %) vs. 42,6 % (95 %-KI 32,2–53,4 %; [18]). Während eine frühere randomisierte Studie sowohl mittel- als auch langfristig Vorteile der Kono-S-Anastomose gegenüber der konventionellen Seit-zu-Seit-Anastomose zeigte, ergibt eine aktuell vorgestellte umfangreichere multizentrische, randomisierte Studie mit einer „side-to-side functional end anastomosis“ keine signifikanten Gruppenunterschiede (25,9 % vs. 27,8 %; [19, 20]). Weitere Studien sind notwendig. Erfreulicherweise werden die Hypothesen, dass handgenähte Anastomosen besser funktionieren als maschinell gestapelte und dass End-zu-End-Anastomosen besser sind als sackförmige Kono-S-Rekonstruktionen, demnächst in Studien überprüft [21].

## Restaurative Proktokolektomie

Die Proktokolektomie mit ileoanaler Pouch-Anastomose ist die chirurgische Behandlung der CU und wird bei therapierefraktärer Erkrankung, endoskopisch nicht behandelbaren hochgradigen Dysplasien oder kolorektalem Karzinom durchgeführt. Die Operation wird meist 2- oder 3-zeitig durchgeführt mit initialer Kolektomie mit protektiver Ileostomaanlage, gefolgt von ileoanaler Pouchanlage nach 3-6 Monaten und Ileostomarückverlagerung (Abb. 3). Die Betroffenen haben postoperativ eine erhöhte Stuhlfrequenz (ca. 7 pro Tag) aber meist sehr gute funktionelle Ergebnisse bei sehr guter Lebensqualität. Die Rate der chirurgischen Behandlungen bei CU hat über die letzten Dekaden abgenommen. Den Biologika wird dabei eine besondere Rolle zugeschrieben. Im Gegensatz zu Patienten mit MC (siehe oben) scheint jedoch die frühere Therapie mit Biologika (innerhalb 3 Jahren nach Erstdiagnose oder Top-down-Behandlung) im Vergleich zu einer späteren Behandlung (> 3 Jahre nach Erstdiagnose oder Step-up-Behandlung) bei CU zu einer erhöhten Operationswahrscheinlichkeit zu führen (OR 2,86; 95 %-KI 1,3–6,3; [13]).

Operationstechnisch gilt nach wie vor, dass die belassene Rektummukosa nicht länger als 2 cm sein sollte. Vor etwa zehn Jahren erfolgten bei technisch limitierten, schwierigen Konstellationen die ersten mittels transanaler MIC durchgeführten restaurativen Proktokolektomien bei CU [22]. Dieser MIC-Zugang ist in Expertenhand mittlerweile etabliert und erweitert zusammen mit den verschiedenen Anastomosentechniken (Handnaht, „single“ und „double stapling“) die personalisierten Behandlungsmöglichkeiten [23–25].

## Intraoperative Bildgebung

**Abb. 4:**
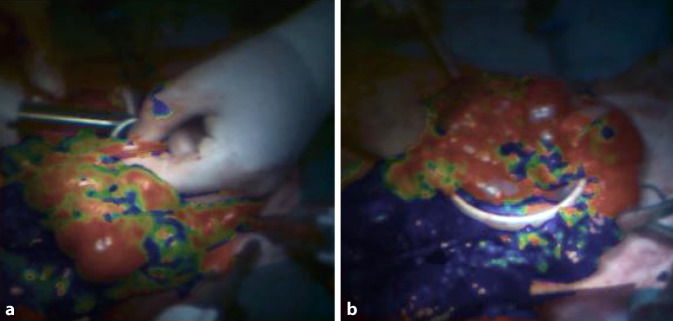
Multispektrale Bildgebung: Datenerfassung bei Ileozökalresektion (M. Crohn). **a** Darstellung der Oxygenierung vor der Durchtrennung des Mesenteriums. **b** Ischämie (Blaufärbung) nach Durchtrennung des Mesenteriums (Forschungsprojekt: Neue Sensorik für multispektrale Bildgebung (NEOSPEK) [27])

Die bereits in der chirurgischen Praxis angekommene Indocyaningrün-Fluoreszenzangiographie kann intraoperativ den Blutfluss visualisieren und kompromittierten Blutfluss anzeigen. So kann intraoperativ die Gewebedurchblutung optimiert werden – mit dem Potential der Verbesserung des chirurgischen Ergebnisses und der Vermeidung von Komplikationen wie Stenosen oder Insuffizienzen. Eine umfangreiche Metaanalyse von 66 Studien mit 11.560 Patienten zeigt, dass der Einsatz der Indocyaningrün-Fluoreszenzangiographie, hyperspektralen Bildgebung oder Laser-speckle-Kontrastbildgebung in der kolorektalen Chirurgie mit einer signifikanten Verringerung von Anastomoseninsuffizienzen verbunden ist [26]. Intraoperativ ist das Unterscheiden von krankem und gesundem Gewebe oft schwierig, dabei könnten jedoch bildgebende Verfahren helfen, die auch geringe Farbunterschiede deutlich visualisieren. Um die klinische Umsetzung der vielversprechenden hyperspektralen und multispektralen Bildgebung voranzutreiben, sind auch Datensätze von Patienten mit CED hilfreich (Abb. 4). Zusammen mit künstlicher Intelligenz kann das Gewebe in Echtzeit analysiert („surgical optomics“) und der Arbeitsablauf für die Ileozökalresektion oder Pouch-Konstruktionen zukünftig erleichtert und sicherer gemacht werden [27–29]).

## Sarkopenie

Sarkopenie, der Verlust von Muskelmasse und Muskelkraft (z. B. Händedruck), ist ein häufiges Symptom bei CED Patienten. Das Auftreten einer Sarkopenie gilt als Hinweis auf ein Therapieversagen. Die Sarkopenie führt eher nicht zur Umstellung der medikamentösen Therapie, sondern gehäuft zur Operationsindikation (OR 1,5; 95 %-KI 1,1–2,2; *p* = 0,023). Für über 60-jährige Patienten gilt sie – wie auch die präoperative Sepsis und eine vorbekannte CED-assoziierte Operation – als unabhängiger Risikofaktor für einen komplikativen postoperativen Verlauf [30, 31]. Leitliniengemäß sollte vor elektiven Operationen ein Screening und gegebenenfalls die Prähabilitation erfolgen, um die operativen Ergebnisse zu verbessern.

## Therapeutische Appendektomie

Die Appendix hat eine immunmodulatorische Rolle und verschiedene Analysen postulieren eine Reduktion der Frequenz der Krankheitsschübe einer Colitis ulcerosa bzw. eine geringere Schwere der Schübe nach Appendektomie. Dies konnte bisher jedoch noch nicht überzeugend nachgewiesen werden. Eine Indikation zur therapeutischen Appendektomie gibt es nach den aktuellen Leitlinienempfehlungen der Deutschen Gesellschaft für Gastroenterologie, Verdauungs- und Stoffwechselkrankheiten (DGVS) daher nicht. Unter der Prämisse des Organ- und Lebensqualitätserhalts kann die Appendektomie bei Patienten mit linksseitiger behandlungsrefraktärer CU im Board weiterhin diskutiert werden. Weitere Daten für diese eigentlich attraktive Behandlungsstrategie wären jedoch wünschenswert und die Ergebnisse der nicht-randomisierten, multizentrischen, prospektiven Kohortenstudie COlonic Salvage by Therapeutic Appendectomy (COSTA), die Patienten mit aktiver CU trotz optimierter Step-up-Behandlung einschließt, bleiben abzuwarten [32, 33]. Weiterhin könnte die randomisierte ACCURE-Studie [34] Aufschluss darüber geben, ob die zusätzliche Appendektomie der alleinigen Erhaltungstherapie in Bezug auf die Aufrechterhaltung der Remission überlegen ist.

## Fazit für die Praxis

- Die chirurgische Behandlung von Patienten mit chronisch-entzündlichen Darmerkrankungen (CED) orientiert sich an Leitlinien. Ihre Komplexität ist hoch.
- Die Wahl des minimal-invasiven Zugangs und der entsprechenden Methode sowie die Wahl der Anastomosentechnik basiert auf der aktuellen wissenschaftlichen Evidenzlage und der Erfahrung des Behandlers.
- Operationstechnische Weiterentwicklungen, beispielsweise in der Robotik und Bildgebung, sollten analog zu den fortschrittlichen medikamentösen Strategien in den CED-Boards Beachtung finden. Sie können somit dynamisch, evidenzbasiert und partizipativ in die multimodalen Konzepte einfließen.
- Die Proktokolektomie ist die chirurgische Therapie der Colitis ulcerosa und wird nicht selten dreizeitig durchgeführt. Postoperativ haben die Patienten eine erhöhte Stuhlfrequenz, aber in der Mehrheit der Fälle gute bis sehr gute Darmfunktion und Lebensqualität.
- Bei Morbus Crohn und isoliertem Befall der Ileozökalklappe ist die Operation der medikamentösen Therapie mindestens gleichwertig.
- Therapieempfehlungen sollten gemeinsam in einem interdisziplinären CED-Board getroffen werden unter früher Einbeziehung der Chirurgie.

## Supplementary Information

Video 1: Rekonstruktion der CT-Bildgebung zur Darstellung und Dichtigkeitsprüfung des kontrastmittelgefüllten ileoanalen J-Pouches vor Ileostomarückverlegung (Fall aus Abbildung 2)
